# (*E*)-4-Meth­oxy-*N*′-(4-nitro­benzyl­idene)benzohydrazide methanol monosolvate

**DOI:** 10.1107/S160053681004701X

**Published:** 2010-11-20

**Authors:** Hong-Yan Ban

**Affiliations:** a School of Chemical Engineering, University of Science and Technology Liaoning, Anshan 114051, People’s Republic of China

## Abstract

The hydrazone mol­ecule of the title compound, C_15_H_13_N_3_O_4_·CH_4_O, is nearly planar, with a dihedral angle between the two benzene rings of 1.2 (4)°. The mol­ecule exists in a *trans* configuration with respect to the central methyl­idene unit. In the crystal, the benzohydrazide and methanol mol­ecules are linked through inter­molecular O—H⋯O, O—H⋯N and N—H⋯O hydrogen bonds, forming chains along the *a* axis.

## Related literature

For the biological activity of hydrazones, see: Zhong *et al.* (2007 ▶); Raj *et al.* (2007 ▶); Jimenez-Pulido *et al.* (2008 ▶). For related structures, see: Ban & Li (2008*a* ▶,*b* ▶); Li & Ban (2009*a* ▶,*b* ▶); Yehye *et al.* (2008 ▶); Fun, Patil, Jebas *et al.*, 2008 ▶; Fun, Patil, Rao *et al.*, 2008 ▶; Yang *et al.* (2008 ▶); Ejsmont *et al.* (2008 ▶).
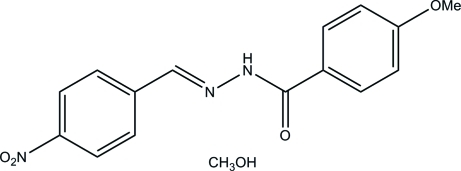


## Experimental

### 

#### Crystal data

C_15_H_13_N_3_O_4_·CH_4_O
*M* *_r_* = 331.33
Monoclinic, 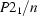

*a* = 6.6482 (14) Å
*b* = 17.730 (3) Å
*c* = 13.898 (2) Å
β = 95.004 (3)°
*V* = 1631.9 (5) Å^3^
*Z* = 4
Mo *K*α radiation
μ = 0.10 mm^−1^
*T* = 298 K
0.20 × 0.17 × 0.17 mm

#### Data collection

Bruker SMART CCD area-detector diffractometer
Absorption correction: multi-scan (*SADABS*; Sheldrick, 2008 ▶) *T*_min_ = 0.980, *T*_max_ = 0.983
12876 measured reflections
3466 independent reflections
1184 reflections with *I* > 2σ(*I*)
*R*_int_ = 0.115

#### Refinement

*R*[*F* ^2^ > 2σ(*F* ^2^)] = 0.081
*wR*(*F* ^2^) = 0.204
*S* = 0.94
3466 reflections
222 parameters
1 restraint
H atoms treated by a mixture of independent and constrained refinement
Δρ_max_ = 0.23 e Å^−3^
Δρ_min_ = −0.27 e Å^−3^

### 

Data collection: *SMART* (Bruker, 1998 ▶); cell refinement: *SAINT* (Bruker, 1998 ▶); data reduction: *SAINT*; program(s) used to solve structure: *SHELXS97* (Sheldrick, 2008 ▶); program(s) used to refine structure: *SHELXL97* (Sheldrick, 2008 ▶); molecular graphics: *SHELXTL* (Sheldrick, 2008 ▶); software used to prepare material for publication: *SHELXTL*.

## Supplementary Material

Crystal structure: contains datablocks global, I. DOI: 10.1107/S160053681004701X/rz2522sup1.cif

Structure factors: contains datablocks I. DOI: 10.1107/S160053681004701X/rz2522Isup2.hkl

Additional supplementary materials:  crystallographic information; 3D view; checkCIF report

## Figures and Tables

**Table 1:** Hydrogen-bond geometry (Å, °)

*D*—H⋯*A*	*D*—H	H⋯*A*	*D*⋯*A*	*D*—H⋯*A*
O5—H5⋯O3	0.82	2.03	2.812 (4)	159
O5—H5⋯N2	0.82	2.61	3.194 (4)	129
N3—H3*A*⋯O5^i^	0.90 (1)	2.02 (2)	2.900 (4)	166 (4)

Symmetry code: (i) 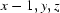.

**Fig. 1.**
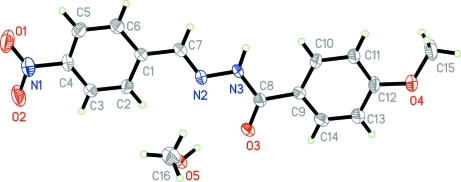
The molecular structure of the title compound, showing 30% probability displacement ellipsoids for the non-hydrogen atoms.

**Fig. 2.**
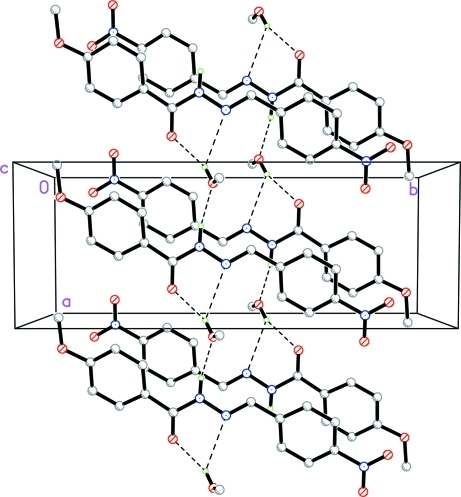
The packing diagram of the title compound, viewed along the c axis. Hydrogen bonds are shown as dashed lines. Hydrogen atoms not involved in hydrogen bonding are omitted for clarity.

## supplementary crystallographic information

### Comment

Hydrazone compounds derived from the condensation of aldehydes with hydrazides have been demonstrated to possess excellent biological activities (Zhong *et al.*, 2007; Raj *et al.*, 2007; Jimenez-Pulido *et al.*, 2008). Due to the easy synthesis of such compounds, a large number of hydrazone compounds have been synthesized and structurally characterized (Yehye *et al.*, 2008; Fun, Patil, Jebas *et al.*, 2008; Fun, Patil, Rao *et al.*, 2008; Yang *et al.*, 2008; Ejsmont *et al.*, 2008). Recently, we have reported a few such compounds (Ban & Li, 2008*a*,*b*; Li & Ban, 2009*a*,*b*). Herein the crystal structure of the title new compound is reported.

The asymmetric unit of the title compound consists of a hydrazone molecule and a methanol molecule (Fig. 1). The hydrazone molecule is nearly planar, the dihedral angle between the two benzene rings being 1.2 (4)°. The molecule exists in a *trans* configuration with respect to the central methylidene unit. In the crystal structure, the hydrazone molecules and the methanol molecules are linked through intermolecular O—H···O, O—H···N and N—H···O hydrogen bonds (Table 1), forming chains along the *a* axis (Fig. 2).

### Experimental

The title compound was prepared by refluxing 4-nitrobenzaldehyde (1.0 mol) with 4-methoxybenzohydrazide (1.0 mol) in methanol (100 ml). Excess methanol was removed from the mixture by distillation. A colourless solid product was filtered, and washed three times with methanol. Colourless block-shaped crystals of the title compound were obtained from a methanol solution by slow evaporation in air.

### Refinement

Atom H3A was located in a difference Fourier map and refined isotropically, with the N—H distance restrained to 0.90 (1)Å and *U*_iso_ fixed at 0.08 Å^2^. The remaining H atoms were placed in calculated positions (C—H = 0.93–0.96 Å and O—H = 0.82 Å) and refined as riding with *U*_iso_(H) = 1.2*U*_eq_(C) or 1.5*U*_eq_(O and methyl C).

### Crystal data

**Table:**

C_15_H_13_N_3_O_4_·CH_4_O	*F*(000) = 696
*M**_r_* = 331.33	*D*_x_ = 1.349 Mg m^−^^3^
Monoclinic, *P*2_1_/*n*	Mo *K*α radiation, λ = 0.71073 Å
Hall symbol: -P 2yn	Cell parameters from 794 reflections
*a* = 6.6482 (14) Å	θ = 2.7–26.5°
*b* = 17.730 (3) Å	µ = 0.10 mm^−^^1^
*c* = 13.898 (2) Å	*T* = 298 K
β = 95.004 (3)°	Block, colourless
*V* = 1631.9 (5) Å^3^	0.20 × 0.17 × 0.17 mm
*Z* = 4	

### Data collection

**Table:**

Bruker SMART CCD area-detector diffractometer	3466 independent reflections
Radiation source: fine-focus sealed tube	1184 reflections with *I* > 2σ(*I*)
graphite	*R*_int_ = 0.115
ω scans	θ_max_ = 27.0°, θ_min_ = 1.9°
Absorption correction: multi-scan (*SADABS*; Sheldrick, 2008)	*h* = −8→8
*T*_min_ = 0.980, *T*_max_ = 0.983	*k* = −22→22
12876 measured reflections	*l* = −17→17

### Refinement

**Table:**

Refinement on *F*^2^	Primary atom site location: structure-invariant direct methods
Least-squares matrix: full	Secondary atom site location: difference Fourier map
*R*[*F*^2^ > 2σ(*F*^2^)] = 0.081	Hydrogen site location: inferred from neighbouring sites
*wR*(*F*^2^) = 0.204	H atoms treated by a mixture of independent and constrained refinement
*S* = 0.94	*w* = 1/[σ^2^(*F*_o_^2^) + (0.0757*P*)^2^] where *P* = (*F*_o_^2^ + 2*F*_c_^2^)/3
3466 reflections	(Δ/σ)_max_ < 0.001
222 parameters	Δρ_max_ = 0.23 e Å^−^^3^
1 restraint	Δρ_min_ = −0.27 e Å^−^^3^

### Special details

**Table:**

Geometry. All e.s.d.'s (except the e.s.d. in the dihedral angle between two l.s. planes) are estimated using the full covariance matrix. The cell e.s.d.'s are taken into account individually in the estimation of e.s.d.'s in distances, angles and torsion angles; correlations between e.s.d.'s in cell parameters are only used when they are defined by crystal symmetry. An approximate (isotropic) treatment of cell e.s.d.'s is used for estimating e.s.d.'s involving l.s. planes.
Refinement. Refinement of *F*^2^ against ALL reflections. The weighted *R*-factor *wR* and goodness of fit *S* are based on *F*^2^, conventional *R*-factors *R* are based on *F*, with *F* set to zero for negative *F*^2^. The threshold expression of *F*^2^ > σ(*F*^2^) is used only for calculating *R*-factors(gt) *etc*. and is not relevant to the choice of reflections for refinement. *R*-factors based on *F*^2^ are statistically about twice as large as those based on *F*, and *R*- factors based on ALL data will be even larger.

### Fractional atomic coordinates and isotropic or equivalent isotropic displacement parameters (Å^2^)

**Table:**

	*x*	*y*	*z*	*U*_iso_*/*U*_eq_	
N1	0.4536 (7)	−0.3135 (2)	0.1424 (3)	0.0634 (12)	
N2	0.1193 (5)	0.0260 (2)	0.1132 (2)	0.0453 (9)	
N3	0.0063 (5)	0.0908 (2)	0.1112 (3)	0.0460 (9)	
O1	0.3670 (6)	−0.3698 (2)	0.1665 (3)	0.0923 (13)	
O2	0.6283 (6)	−0.3132 (2)	0.1217 (3)	0.0930 (13)	
O3	0.2843 (4)	0.15868 (16)	0.0864 (2)	0.0650 (10)	
O4	−0.3188 (4)	0.43167 (16)	0.0795 (2)	0.0709 (10)	
O5	0.5933 (4)	0.05685 (17)	0.1482 (3)	0.0642 (10)	
H5	0.4898	0.0767	0.1237	0.096*	
C1	0.1378 (6)	−0.1068 (2)	0.1293 (3)	0.0424 (11)	
C2	0.3344 (6)	−0.1112 (2)	0.1018 (3)	0.0529 (12)	
H2	0.3964	−0.0682	0.0801	0.063*	
C3	0.4373 (6)	−0.1786 (3)	0.1064 (3)	0.0549 (13)	
H3	0.5680	−0.1817	0.0876	0.066*	
C4	0.3437 (7)	−0.2407 (2)	0.1391 (3)	0.0493 (12)	
C5	0.1521 (7)	−0.2403 (3)	0.1657 (3)	0.0586 (13)	
H5A	0.0922	−0.2840	0.1866	0.070*	
C6	0.0480 (6)	−0.1719 (3)	0.1607 (3)	0.0563 (13)	
H6	−0.0834	−0.1699	0.1786	0.068*	
C7	0.0276 (6)	−0.0354 (3)	0.1262 (3)	0.0495 (12)	
H7	−0.1100	−0.0350	0.1338	0.059*	
C8	0.1041 (7)	0.1569 (2)	0.0971 (3)	0.0461 (11)	
C9	−0.0195 (6)	0.2263 (2)	0.0959 (3)	0.0449 (11)	
C10	−0.2157 (6)	0.2311 (2)	0.1201 (3)	0.0508 (12)	
H10	−0.2785	0.1877	0.1403	0.061*	
C11	−0.3223 (6)	0.2981 (2)	0.1153 (3)	0.0535 (12)	
H11	−0.4551	0.2998	0.1314	0.064*	
C12	−0.2287 (7)	0.3619 (2)	0.0864 (3)	0.0540 (12)	
C13	−0.0345 (7)	0.3593 (3)	0.0605 (4)	0.0829 (18)	
H13	0.0277	0.4028	0.0402	0.100*	
C14	0.0666 (7)	0.2920 (3)	0.0649 (4)	0.0745 (16)	
H14	0.1977	0.2904	0.0465	0.089*	
C15	−0.5263 (8)	0.4384 (3)	0.0967 (4)	0.0833 (17)	
H15A	−0.6078	0.4115	0.0477	0.125*	
H15B	−0.5642	0.4907	0.0951	0.125*	
H15C	−0.5467	0.4177	0.1589	0.125*	
C16	0.5721 (7)	0.0414 (3)	0.2467 (4)	0.0823 (17)	
H16A	0.6181	−0.0089	0.2617	0.123*	
H16B	0.4327	0.0459	0.2588	0.123*	
H16C	0.6512	0.0767	0.2863	0.123*	
H3A	−0.128 (2)	0.087 (2)	0.116 (3)	0.080*	

### Atomic displacement parameters (Å^2^)

**Table:**

	*U*^11^	*U*^22^	*U*^33^	*U*^12^	*U*^13^	*U*^23^
N1	0.077 (3)	0.054 (3)	0.060 (3)	0.021 (3)	0.006 (2)	0.000 (2)
N2	0.041 (2)	0.038 (2)	0.056 (2)	0.0098 (19)	0.0024 (17)	0.0028 (18)
N3	0.030 (2)	0.039 (2)	0.069 (3)	0.0102 (19)	0.0049 (19)	0.0039 (19)
O1	0.117 (3)	0.047 (2)	0.115 (3)	0.022 (2)	0.025 (2)	0.015 (2)
O2	0.078 (3)	0.077 (3)	0.128 (3)	0.043 (2)	0.031 (2)	0.021 (2)
O3	0.0322 (17)	0.053 (2)	0.111 (3)	0.0070 (15)	0.0143 (17)	0.0145 (18)
O4	0.056 (2)	0.0407 (19)	0.115 (3)	0.0141 (17)	−0.0002 (19)	−0.0034 (19)
O5	0.0343 (18)	0.056 (2)	0.102 (3)	0.0096 (16)	0.0027 (17)	0.017 (2)
C1	0.037 (3)	0.040 (3)	0.049 (3)	0.002 (2)	0.004 (2)	0.001 (2)
C2	0.050 (3)	0.036 (3)	0.073 (3)	0.004 (2)	0.013 (2)	0.000 (2)
C3	0.037 (3)	0.050 (3)	0.077 (4)	0.005 (2)	0.006 (2)	0.002 (3)
C4	0.058 (3)	0.042 (3)	0.048 (3)	0.017 (2)	0.003 (2)	0.003 (2)
C5	0.061 (3)	0.044 (3)	0.073 (3)	−0.002 (3)	0.017 (3)	0.008 (2)
C6	0.048 (3)	0.052 (3)	0.070 (4)	0.007 (3)	0.017 (2)	0.002 (3)
C7	0.034 (2)	0.051 (3)	0.063 (3)	0.007 (2)	0.004 (2)	0.002 (2)
C8	0.042 (3)	0.044 (3)	0.053 (3)	0.007 (2)	0.001 (2)	0.009 (2)
C9	0.035 (3)	0.044 (3)	0.056 (3)	0.001 (2)	−0.001 (2)	0.002 (2)
C10	0.052 (3)	0.031 (3)	0.071 (3)	0.003 (2)	0.010 (2)	0.007 (2)
C11	0.049 (3)	0.039 (3)	0.074 (3)	0.008 (2)	0.013 (2)	0.009 (2)
C12	0.059 (3)	0.034 (3)	0.067 (3)	0.013 (2)	−0.005 (3)	0.002 (2)
C13	0.051 (3)	0.047 (3)	0.152 (5)	0.005 (3)	0.020 (3)	0.015 (3)
C14	0.037 (3)	0.057 (3)	0.131 (5)	0.003 (3)	0.012 (3)	0.020 (3)
C15	0.091 (4)	0.057 (3)	0.106 (4)	0.036 (3)	0.029 (3)	0.009 (3)
C16	0.069 (4)	0.082 (4)	0.094 (5)	0.006 (3)	−0.009 (3)	−0.002 (3)

### Geometric parameters (Å, °)

**Table:**

N1—O1	1.214 (4)	C5—H5A	0.9300
N1—O2	1.221 (5)	C6—H6	0.9300
N1—C4	1.482 (5)	C7—H7	0.9300
N2—C7	1.269 (5)	C8—C9	1.479 (5)
N2—N3	1.372 (4)	C9—C10	1.377 (5)
N3—C8	1.363 (5)	C9—C14	1.383 (5)
N3—H3A	0.902 (10)	C10—C11	1.383 (5)
O3—C8	1.221 (4)	C10—H10	0.9300
O4—C12	1.374 (5)	C11—C12	1.367 (5)
O4—C15	1.426 (5)	C11—H11	0.9300
O5—C16	1.415 (5)	C12—C13	1.371 (6)
O5—H5	0.8200	C13—C14	1.369 (6)
C1—C6	1.386 (5)	C13—H13	0.9300
C1—C2	1.395 (5)	C14—H14	0.9300
C1—C7	1.461 (5)	C15—H15A	0.9600
C2—C3	1.376 (5)	C15—H15B	0.9600
C2—H2	0.9300	C15—H15C	0.9600
C3—C4	1.362 (5)	C16—H16A	0.9600
C3—H3	0.9300	C16—H16B	0.9600
C4—C5	1.357 (5)	C16—H16C	0.9600
C5—C6	1.395 (5)		
			
O1—N1—O2	123.5 (4)	N3—C8—C9	116.5 (4)
O1—N1—C4	118.7 (4)	C10—C9—C14	116.8 (4)
O2—N1—C4	117.7 (5)	C10—C9—C8	125.8 (4)
C7—N2—N3	116.9 (3)	C14—C9—C8	117.4 (4)
C8—N3—N2	117.2 (3)	C9—C10—C11	122.2 (4)
C8—N3—H3A	124 (3)	C9—C10—H10	118.9
N2—N3—H3A	119 (3)	C11—C10—H10	118.9
C12—O4—C15	119.1 (4)	C12—C11—C10	118.7 (4)
C16—O5—H5	109.5	C12—C11—H11	120.7
C6—C1—C2	118.6 (4)	C10—C11—H11	120.6
C6—C1—C7	120.1 (4)	C11—C12—C13	120.9 (4)
C2—C1—C7	121.3 (4)	C11—C12—O4	123.9 (4)
C3—C2—C1	120.6 (4)	C13—C12—O4	115.2 (4)
C3—C2—H2	119.7	C14—C13—C12	119.2 (5)
C1—C2—H2	119.7	C14—C13—H13	120.4
C4—C3—C2	118.5 (4)	C12—C13—H13	120.4
C4—C3—H3	120.7	C13—C14—C9	122.2 (4)
C2—C3—H3	120.7	C13—C14—H14	118.9
C5—C4—C3	123.6 (4)	C9—C14—H14	118.9
C5—C4—N1	118.0 (4)	O4—C15—H15A	109.5
C3—C4—N1	118.4 (4)	O4—C15—H15B	109.5
C4—C5—C6	117.7 (4)	H15A—C15—H15B	109.5
C4—C5—H5A	121.2	O4—C15—H15C	109.5
C6—C5—H5A	121.2	H15A—C15—H15C	109.5
C1—C6—C5	120.9 (4)	H15B—C15—H15C	109.5
C1—C6—H6	119.5	O5—C16—H16A	109.5
C5—C6—H6	119.5	O5—C16—H16B	109.5
N2—C7—C1	120.1 (4)	H16A—C16—H16B	109.5
N2—C7—H7	120.0	O5—C16—H16C	109.5
C1—C7—H7	120.0	H16A—C16—H16C	109.5
O3—C8—N3	121.7 (4)	H16B—C16—H16C	109.5
O3—C8—C9	121.8 (4)		

### Hydrogen-bond geometry (Å, °)

**Table:**

*D*—H···*A*	*D*—H	H···*A*	*D*···*A*	*D*—H···*A*
O5—H5···O3	0.82	2.03	2.812 (4)	159
O5—H5···N2	0.82	2.61	3.194 (4)	129
N3—H3A···O5^i^	0.90 (1)	2.02 (2)	2.900 (4)	166 (4)

Symmetry codes: (i) *x*−1, *y*, *z*.
